# Mortality and Incidence Rates of Stomach Cancer in the JACC Study

**DOI:** 10.2188/jea.15.S89

**Published:** 2005-08-18

**Authors:** Pham Truong Minh, Yoshihisa Fujino, Takesumi Yoshimura, Noritaka Tokui, Tetsuya Mizoue, Hiroshi Yatsuya, Hideaki Toyoshima, Kiyomi Sakata, Shogo Kikuchi, Yoshihiro Hoshiyama, Tatsuhiko Kubo, Akiko Tamakoshi

**Affiliations:** 1 Department of Clinical Epidemiology, Institute of Industrial Ecological Sciences, University of Occupational and Environmental Health.; 2 Fukuoka Institute of Occupational Health.; 3 Fukuoka Institute of Health and Environmental Sciences.; 4 Department of Preventive Medicine, Graduate School of Medical Sciences, Kyushu University.; 5 Department of Public Health/Health Information Dynamics, Nagoya University Graduate School of Medicine.; 6 Department of Public Health, Wakayama Medical University.; 7 Department of Public Health, Aichi Medical University School of Medicine.; 8 Department of Public Health, Showa University School of Medicine.; 9 Department of Preventive Medicine/Biostatistics and Medical Decision Making, Nagoya University Graduate School of Medicine.

**Keywords:** JACC Study, Stomach Neoplasms, Incidence, Mortality

## Abstract

BACKGROUND: The objective of this study was to examine the mortality and incidence of stomach cancer in the Japan Collaborative Cohort Study (JACC Study), and compare them with those of the general population of Japan.

METHODS: The cohort study involved 127,477 people living in 45 municipalities in Japan. The deaths due to stomach cancer were ascertained by death certificates. The age-adjusted mortality rate per 100,000 was calculated based on 110,792 subjects aged between 40 and 79 years at the baseline in all study areas. The incident cases were defined as the subjects in whom stomach cancer had developed, or subjects who died of stomach cancer during the observed period of survey for cancer incidence. The age-adjusted incidence rate per 100,000 was ascertained and calculated in 64,820 people aged 40-79 years at the baseline living in 24 study municipalities with cancer registries. Then, the mortality rate was compared with the mortality data published in vital statistics of Japan of 1995, and the incidence rate compared with the incidence data published in The Research Group for Population-based Cancer Registration in Japan of 1992.

RESULTS: During more than 10 years of follow-up, there were 582 male deaths and 287 female deaths due to stomach cancer. The age-adjusted mortality rate was 93.4/100,000 person-year (95% confidence interval [CI]: 84.6-102.2) in males and 31.1 (95% CI: 27.0-35.3) in females. There were 646 incident cases in males and 370 in females. The age-adjusted incidence rate was 245.3 / 100,000 person-year (95% CI: 221.6-268.9) and 94.8 (95% CI: 83.0-106.6) in males and females, respectively.

CONCLUSION: The mortality rate of stomach cancer in the JACC Study was lower than that in the vital statistics in Japan. Similar incidence rate of stomach cancer seems to be found between data of the JACC Study and that of the Research Group for Population-based Cancer Registration, but care is needed to interpret this similarity, because it might be due to different degree of completeness of incidence survey between the 2 studies

Stomach cancer is the second most common cause of cancer deaths in the world.^1^ However, in recent decades, its incidence and mortality have declined markedly in most industrialized countries. In spite of this favorable trend, Japan has been considered as one of the countries where stomach cancer mortality as well as incidence remains the highest in the world,^2^ and stomach cancer is still one of the major causes of death. Remarkable changes in lifestyle and living conditions from around the 1960’s contributed to the changing health pattern in general and stomach cancer in particular for the Japanese. Several cohort studies have been done to evaluate the cancer risk in Japan such as the Hirayama study conducted in 6 prefectures in Japan (Miyagi, Aichi, Osaka, Hyogo, Okayama, and Kagoshima),^3^ which was started in 1965 and terminated in 1982, and the other cohort studies conducted in 1990 in Miyagi Prefecture,^4^ and the Japan Public Health Center-based prospective Study on Cancer and Cardiovascular diseases.^5^

The nation-wide study, the Japan Collaborative Cohort Study (JACC Study) for Evaluation of Cancer Risk sponsored by the Ministry of Education, Science, Sports and Culture of Japan (Monbusho), was one of the cohort studies conducted for the purpose of demonstrating the risk factors and all cancers, including stomach cancer. The JACC Study has reported several findings that might be potentially beneficial for the prevention against stomach cancer for the Japanese population. It was characterized by the fact that the subjects were collected throughout Japan. However, the areas and the subjects that were involved in the JACC Study were not randomly selected, which means that the subjects in the JACC Study are not necessarily representative of a general population of Japan. Therefore, it is particularly important to know the mortality and incidence of stomach cancer in the JACC Study in order to appraise the knowledge that has been obtained from this large-scale cohort. Recently, Watanabe^6^ reported standardized mortality ratio of stomach cancer in the JACC Study, but incidence of stomach cancer was not yet reported. So that, in this paper we further examined both the mortality and incidence of stomach cancer in the JACC Study, by the direct method of adjustment, and compared them with those of the general population of Japan.

## METHODS

### Study population

The JACC Study was established to identify and evaluate various risks or protective factors of all cancers. This study has been described elsewhere.^7^ A total of 45 municipalities were involved in this prospective study, but they were not randomly selected. They included 6 cities, 34 towns and 5 villages scattered throughout Japan. Among them, study participants came from the entire population in 22 municipalities, and from health screening subjects in 23 municipalities. Study places were divided into 7 regions namely Hokkaido, Tohoku, Kanto, Chubu, Kinki, Chugoku and Kyushu region. Initially, the JACC Study involved 127,477 inhabitants of the areas, who responded to the questionnaire in 1988-1990, as a basic cohort population. Then the subjects were restricted to 110,792 people (46,465 males and 64,327 females), aged from 40 to 79 years old at the entry, in which most type of cancers commonly occurred. The JACC Study originally aimed to investigate variety of risk factors associated with any kind of cancer.

To evaluate stomach cancer mortality rate, 110,792 subjects in all study areas were available. The vital status of all study participants were checked annually until December 31, 1999. The subjects who moved out of the study areas were annually verified by reviewing the population registries of the cohort participants, and were treated as censored cases. The subjects who died during the study were ascertained by reviewing all death certificates in each area once a year under the authoritative permission from the Director General of the Prime Minister’s Office (Ministry of Public Management, Home Affairs, Post and Telecommunication). All causes of death were coded based on the International Classification of Diseases and Injuries (ICD), 9th Revision (ICD-9) from baseline surveys through the end of 1994, and 10th Revision (ICD-10) in and after 1995. Stomach cancer was coded from 151.0 to 151.9 (ICD-9) or from C16.0 to C16.9 (ICD-10). All ICD-9 codes were converted into ICD-10 codes for the analysis.

To evaluate stomach cancer incidence rate, the procedure of follow up was the same as in evaluating stomach cancer mortality. The incidence of cancer was ascertained only in 24 study municipalities (among 45 municipalities), where cancer registries were available, and we called these areas as areas of survey for the incidence. The source of cancer registries were from population-based cancer registries in 20 municipalities and from hospital-based cancer registries in 4 others. The vital status of study participants were checked annually to identify the subjects who had moved out, who had died, or were still alive. In addition, the incident cases of stomach cancer were recorded. The ICD-9 was used until the end of 1994 and ICD-10 from 1995, to record all incident cases of stomach cancer, and all ICD-9 codes were converted into ICD-10 codes for the analysis. The end of follow up was December 31, 1994 in one study area because of accidental interruption in the survey, or December 31, 1997 for the remaining study areas. For analysis, the incident cases of stomach cancer were defined as the subjects in whom stomach cancer had developed, or subjects who died from stomach cancer during the observed period of survey for cancer incidence. Because some stomach cancer cases could not be reported at the time of diagnosis, but the time of death, we counted those cases as incident cases for calculation of incidence. After excluding the subjects who had stomach cancer before the baseline surveys and excluding the subjects of age under 40 years, or older than 79 years at the baseline, the remaining 64,820 people (26,229 males and 38,591 females) were available for calculation of stomach cancer incidence. To evaluate completeness of survey for stomach cancer incidence, we used the ratio between number of deaths due to stomach cancer and the number of incident cases of stomach cancer (DI ratio). When the DI ratio exceeds 0.7, it is considered as under-registration^8^ of stomach cancer incidence.

Our entire study design, which comprised singular and collective use of epidemiologic data, was approved in 2000 by the Ethical Board at Nagoya University School of Medicine, where the central secretariat of the JACC Study is located, and study design for stomach cancer was also approved by the Ethics Committee of Medical Care and Research, University of Occupational and Environmental Health, Kitakyushu.

### Statistics

We calculated the mortality rate per 100,000 as well as incidence rate per 100,000 based on the calendar year. We categorized age into 5-year age groups of 40-44, 45-49, 50-54, 55-59, 60-64, 65-69, 70-74, 75-79, 80-84 and 85+ groups, to present age-specific mortality rate and age-specific incidence rate. We considered age variable as a time-dependent variable. Naturally, after one year of follow-up, all the subjects get 1 year older than the previous year. Therefore, age group had to be reclassified every year, up to the last year of study. The age-adjusted mortality rate per 100,000 and age-adjusted incidence rate per 100,000 were calculated with the direct method, using the Japanese population structure from the 1985 census.

In order to compare the mortality and incidence rates in the JACC Study with the general Japanese population, we picked up the mortality data published in vital statistics of Japan, data in 1995,^9^ and incidence data published in the Research Group for Population-based Cancer Registration in Japan, data of 1992^10^ as reference data because stomach cancer mortality as well as incidence have shown a declining trend over four decades in Japan.^11^ Mortality data of 1995 and incidence data of 1992 were referenced because these years were assumed to be considered around the middle of the cohort study. All statistic calculations in this analysis were performed using Stata^®^ software release 8.2.

## RESULTS

In more than 10 years of follow-up in 110,792 subjects for mortality calculation, there were 1,103,255.9 person-years counted (455,380.6 person-years observed in males and 647,875.3 person-years observed in females). Total cancer deaths were observed in 4,528 people. Among them, 582 male deaths and 287 female deaths were due to stomach cancer. Thus, the proportion of stomach cancer was 19.2% among total deaths due to all kinds of cancers.

Table 1 shows the age-specific mortality rate, crude mortality rate, and age-adjusted mortality rate of stomach cancer in the JACC Study and in the vital statistics in 1995. In the JACC Study, the crude mortality rate was 127.8 / 100,000 person-year in males and 44.3 in females. The age-adjusted mortality rate was 93.4 / 100,000 person-year (95% confidence interval [CI]: 84.6102.2) in males and 31.1 (95% CI: 27.0-35.3) in females. In the vital statistics, age-adjusted mortality rate was 105.5 / 100,000 population (95% CI: 104.3-106.7) in males and 41.4 (95% CI: 40.8-42.1) in females. Most age-specific mortality rates in the JACC Study were lower than those in the vital statistics. The age-adjusted mortality rate for in the JACC Study was lower than those for the vital statistics in both sexes.

**Table 1.** Stomach cancer mortality rate by sex and age.

Age (year)	Japan Collaborative Cohort Study			Mortality rate in Japan (1995)* (/100,000 population)

	No of deaths	Person-years	Mortality rate (/100,000 person-year)	
Males				

40-44	3	18,935.8	15.8	11.0
45-49	9	45,789.3	19.7	19.4
50-54	18	59,622.7	30.2	37.0
55-59	32	67,174.0	47.6	66.7
60-64	74	78,200.1	94.6	112.1
65-69	114	75,227.3	151.5	174.4
70-74	123	55,000.3	223.6	252.1
75-79	103	35,409.7	290.9	364.4
80-84	93	17,919.0	519	495.7
85+	13	2,102.4	618.3	653.7
All	582	455,380.6		
Crude mortality rate			127.8	106.4
Age-adjusted mortality rate** (95% CI)			93.4 (84.6-102.2)	105.5 (104.3-106.7)

Females				

40-44	1	23,272.3	4.3	10.1
45-49	3	59,345.8	5.1	15.8
50-54	9	82,138.6	11	18.4
55-59	14	95,898.2	14.6	26.6
60-64	41	109,440.0	37.5	37.2
65-69	48	106,894.0	44.9	56.4
70-74	55	82,755.9	66.5	85.3
75-79	59	54,713.0	107.8	125.8
80-84	46	29,349.3	156.7	202.1
85+	11	4,068.2	270.4	283.7
All	287	647,875.3		
Crude mortality rate			44.3	51.1
Age-adjusted mortality rate (95%CI)			31.1 (27.0-35.3)	41.4 (40.8-42.1)

* : Vital statistics of Japan in 1995.

** : Using Japanese population 1985 (both sexes).

CI : confidence interval.

Table 2 shows the crude and age-adjusted mortality rates by sex and area. The age-adjusted mortality rate range was from 59.2 to 123.7 / 100,000 person-year in males and 9.2 to 38.7 in females.

**Table 2.** Crude and age-adjusted mortality rate of stomach cancer by sex and area.

Areas	No of subjects	No of deaths	Person-years	Crude mortality rate (/100,000 person-year)	Age-adjusted mortality rate* (95% CI) (/100,000 person-year)
Males					

Hokkaido	1,465	13	14,396.3	90.3	65.5 (26.4-104.6)
Tohoku	6,238	75	59,506.9	126.0	105.5 (73.9-137.1)
Kanto	4,391	57	39,925.9	142.8	111.8 (76.4-147.1)
Chubu	15,720	200	157,036.8	127.4	94.6 (80.2-108.9)
Kinki	8,000	92	75,991.2	121.1	84.7 (65.2-104.3)
Chugoku	5,094	59	47,436.2	124.4	59.2 (42.6- 75.7)
Kyushu	5,557	86	61,087.3	140.8	123.7 (90.6-156.8)
All areas	46,465	582	455,380.6	127.8	93.4 (84.6-102.2)

Females					

Hokkaido	2,258	3	22,380.2	13.4	9.2 ( 1.1- 19.7)
Tohoku	8,795	38	87,241.7	43.6	38.6 (23.4- 53.8)
Kanto	5,704	26	53,276.0	48.8	32.8 (19.9- 45.6)
Chubu	18,136	88	186,822.3	47.1	30.4 (23.7- 37.1)
Kinki	10,150	41	99,249.2	41.3	32.7 (19.5- 46.0)
Chugoku	9,594	39	93,163.9	41.9	21.5 (13.8- 29.2)
Kyushu	9,690	52	105,742.0	49.2	38.7 (27.5- 49.8)
All areas	64,327	287	647,875.3	44.3	33.1 (27.0- 35.3)

* : Using Japanese population 1985 (both sexes).

CI : confidence interval.

In more than 10 years of follow-up in 64,820 subjects for calculation of stomach cancer incidence, there were 203,784.4 person-years observed in males and 297,626.7 person-years observed in females. There were 646 and 370 incident cases of stomach cancer counted in males and females, respectively. Table 3 shows the age-specific, crude incidence, and age-adjusted incidence rates of stomach cancer in the JACC Study and in the Research Group for Population-based Cancer Registration. In the JACC Study, the crude incidence rate was 317.0 / 100,000 person-year in males and 124.3 in females. The age-adjusted incidence rate was 245.3 / 100,000 person-year (95% CI: 221.6-268.9) and 94.8 (95%CI: 83.0-106.6) in males and females, respectively. In the Research Group for Population-based Cancer Registration , age-adjusted incidence rate was 238.3 / 100,000 population (95% CI: 236.5-240.2) and 95.6 (95% CI: 94.5-96.6) in males and females, respectively. In comparison with the Research Group for Population-based Cancer Registration, the age-adjusted incidence rate of the JACC Study was higher in males (245.3 vs. 238.3) and lower in females (94.8 vs. 95.6), but their 95% CI overlapped with those in the Research Group for Population-based Cancer Registration, meaning that the difference was not significant.

**Table 3.** Stomach cancer incidence rate by sex and age.

Age (year)	Japan Collaborative Cohort Study			JRC* in 1992 incidence rate (/100,000 population)

	No of incidences	Person-years	Incidence rate (/100,000 person-year)	
Males				

40-44	6	10,220.2	58.7	45.6
45-49	16	23,527.8	68.0	61.0
50-54	32	26,599.7	120.3	123.2
55-59	42	30,413.6	138.1	198.6
60-64	102	36,457.7	279.8	319.8
65-69	132	31,604.6	417.7	450.1
70-74	145	22,448.9	645.9	529.6
75-79	106	15,412.3	687.8	655.3
80-84	93	6,733.3	905.9	729.9
85+	13	366.3	109.2	730.5
All	646	203,784.4		
Crude incidence rate			317.0	243.8
Age-adjusted incidence rate** (95%CI)			245.3 (221.6-268.9)	238.3 (236.5-240.2)

Females				

40-44	6	12,978.1	46.2	35.8
45-49	8	31,911.8	25.1	35.9
50-54	16	38,251.9	41.8	57.3
55-59	29	45,642.8	63.5	74.0
60-64	64	52,890.3	121	104.7
65-69	66	47,158.6	139.9	146.2
70-74	76	34,378.3	221.1	202.0
75-79	68	22,893.9	297	241.8
80-84	36	10,839.8	332.1	339.8
85+	1	681.2	146.8	318.4
All	370	297,626.7		
Crude incidence rate			124.3	108.4
Age-adjusted incidence rate (95% CI)			94.8 (83.0-106.6)	95.6 (94.5-96.6)

* : The Research Group for Population-based Cancer Registration in Japan.

** : Using Japanese population 1985 (both sexes).

CI : confidence interval.

Table 4 presents the crude and age-adjusted incidence rates of stomach cancer by sex and area. The age-adjusted incidence rates varied from 107.5 to 291.3 / 100,000 person-year in males and from 28.8 to 119.3 in females. The DI ratio shows completeness of survey for stomach cancer incidence, those ratios varied from 0.26 to 0.62 in males and from 0.22 to 0.65 in females (not including Hokkaido).

**Table 4.** Crude and age-adjusted stomach cancer incidence rate by sex and area.

Areas	No of subjects	No of deaths (1)	No of incidences (2)	DI ratio* {(1)/(2)}	Person-years	Crude incidence rate (/100,000 person-year)	Age-adjusted incidence rate** (95% CI) (/100,000 person-year)
Males							

Hokkaido	247	1	2	0.50	1,895.6	105.5	107.5 ( 1.1-262.2)
Tohoku	1,696	23	55	0.42	14,421.8	381.4	291.3 (150.0-387.7)
Kanto	3,105	33	53	0.62	24,989.8	212.1	182.9 (111.2-254.6)
Chubu	11,791	107	368	0.29	96,678.2	380.6	290.6 (259.5-321.8)
Kinki	3,269	16	56	0.29	26,665.0	210.0	165.5 (111.7-219.2)
Chugoku	4,982	24	92	0.26	30,357.0	303.1	233.0 (158.1-308.4)
Kyushu	1,139	6	20	0.30	8,777.2	227.9	190.5 ( 61.4-319.5)
All areas	26,229	210	646	0.33	203,784.6	317.0	245.3 (221.6-268.9)

Females							

Hokkaido	405	0	1	0.00	3,051.9	32.8	28.8 ( 1.2- 85.0)
Tohoku	2,554	13	33	0.39	22,948.2	143.8	87.2 (57.3-117.1)
Kanto	3,802	15	23	0.65	31,848.0	72.2	50.8 (29.6- 72.1)
Chubu	13,699	45	164	0.27	115,721.9	141.7	104.2 (85.6-122.7)
Kinki	4,553	14	36	0.39	37,365.3	96.3	97.0 (51.6-142.4)
Chugoku	9,517	19	86	0.22	55,166.2	155.9	117.8 (60.5-169.1)
Kyushu	4,061	11	27	0.41	31,525.2	85.6	119.3 (30.9-207.8)
All areas	38,591	117	370	0.32	297,626.7	124.3	94.8 (83.0-106.6)

* : Number of deaths / Number of incidences.

** : Using Japanese population 1985 (both sexes).

CI : confidence interval.

The results shows that the ratio between incidence and mortality rates remains over 2 fold in both sexes. The age-adjusted incidence rate was 245.3 and age-adjusted mortality rate was 93.4 in males. The age-adjusted incidence rate was 94.8 and age-adjusted mortality rate was 31.1 in females.

## DISCUSSION

The JACC Study showed a lower mortality rate due to stomach cancer than that of the vital statistics of Japan,^9^ and a similar incidence rate due to stomach cancer compared with the data from population-based cancer registration.^10^ It should be noted that we reported the truncated mortality and incidence rates of stomach cancer from about a 10-year of follow-up in the JACC Study because the JACC Study included subjects aged over 40. Therefore, care is needed to interpret these results when comparing with other reported data including all age ranges. However, we accounted age by means of direct method of adjustment so that the comparisons between the JACC Study and the vital statistics/population cancer registry we made in the present study might be comparable.

In our research, a slightly lower mortality rate of stomach cancer in comparison to those in general population was reported in both sexes, consistent with the paper reported by Watanabe.^6^ There are several possible explanations for this result. Firstly, healthy volunteer effect might be inherent in the JACC Study. Healthy volunteer effect is a self-selection bias that induces the observed lower mortality of the participants than those of the non-participants, which have been reported from several cohort studies.^12^^,^^13^ Secondly, the JACC Study did not guarantee the representativeness of a general population of Japan, although the JACC Study covered wide areas throughout Japan. There is a possibility that the participants came from areas where the mortality of stomach cancer is low.

In the present study, the incidence rate of stomach cancer is similar to the data from the other study, while the mortality rate of stomach cancer is lower than that of the general population of Japan. When we examined the mortality of stomach cancer in the same population as those in which calculation of incidence had been done (i.e. 64,820 people), the age-adjusted mortality rates were 82.7 / 100,000 person-year (95% CI: 71.8-93.6) in males and 30.0 (95% CI: 24.5-35.4) in females (data not shown). Therefore, these results showed a consistently lower mortality in comparison to that in the general population, while the incidence of stomach cancer was close to the data of the Research Group for Population-based Cancer Registration (data in 1992). We have no explanation for this discrepancy between the mortality and incidence, but one possible explanation might be the completeness of incidence survey. We evaluated the completeness of incidence survey by means of DI ratios. DI ratio of stomach cancer in the JACC Study was 0.33 for males and 0.32 for females. According to the data of the Research Group for Population-based Cancer Registration,^14^ their DI ratio for all cancer was 0.55. This means that the survey for incidence of the JACC Study is more accurate than that of the Research Group for Population-based Cancer Registration. Therefore, if the completeness of the Research Group for Population-based Cancer Registration were more accurate, the number of incident cases of the Research Group could be increased, and then the incidence of the JACC Study could be relatively lower than that of the Research Group for Population-based Cancer Registration. Thus, care is needed to interpret similarity of incidence.

We reported the differences in the mortality and incidence of stomach cancer between the areas. It is well known that occurrence of stomach cancer is largely different between areas suggesting that stomach cancer is related to environmental factors that are strongly associated with geographical areas. However, care is needed to interpret the results for each area of the JACC Study because the areas and subjects were not randomly selected, so that the results for each area are not comparable to the incidence and mortality of stomach cancer reported by national statistics.^15^ For example, the area that refers to Kyushu in the present study actually consisted of small parts of Fukuoka and Saga Prefectures. In fact, based on the standardized mortality ratios (data in 2000) listed in the Cancer Statistics in Japan-2003,^15^ Kyushu has been known as the areas with low or moderate level, while the JACC Study showed the highest age-adjusted mortality rate in Kyushu (both sexes).

In conclusion, the mortality rate of stomach cancer in the JACC Study was lower than that in the vital statistics. Similar incidence rate of stomach cancer seems to be found between data of the JACC Study and that of the Research Group for Population-based Cancer Registration, but care is needed to interpret this similarity because it might be due to different degree of completeness of incidence survey between the JACC Study and the Research Group for Population-based Cancer Registration.
